# Anti-Inflammatory Effects of Hyperbaric Oxygenation during DSS-Induced Colitis in BALB/c Mice Include Changes in Gene Expression of* HIF-1α*, Proinflammatory Cytokines, and Antioxidative Enzymes

**DOI:** 10.1155/2016/7141430

**Published:** 2016-08-30

**Authors:** Sanja Novak, Ines Drenjancevic, Rosemary Vukovic, Zoltán Kellermayer, Anita Cosic, Maja Tolusic Levak, Péter Balogh, Filip Culo, Martina Mihalj

**Affiliations:** ^1^Department of Physiology and Immunology, Faculty of Medicine, University of Osijek, 31 000 Osijek, Croatia; ^2^Department of Biology, University of Osijek, 31 000 Osijek, Croatia; ^3^Department of Immunology and Biotechnology, Faculty of Medicine, University of Pécs, 7624 Pécs, Hungary; ^4^Lymphoid Organogenesis Research Group, Szentágothai Research Center, University of Pécs, 7624 Pécs, Hungary; ^5^Department of Histology and Embryology, Faculty of Medicine, University of Osijek, 31 000 Osijek, Croatia

## Abstract

Reactive oxygen species (ROS) and nitrogen species have an indispensable role in regulating cell signalling pathways, including transcriptional control via hypoxia inducible factor-1*α* (*HIF-1α*). Hyperbaric oxygenation treatment (HBO_2_) increases tissue oxygen content and leads to enhanced ROS production. In the present study DSS-induced colitis has been employed in BALB/c mice as an experimental model of gut mucosa inflammation to investigate the effects of HBO_2_ on* HIF-1α*, antioxidative enzyme, and proinflammatory cytokine genes during the colonic inflammation. Here we report that HBO_2_ significantly reduces severity of DSS-induced colitis, as evidenced by the clinical features, histological assessment, impaired immune cell expansion and mobilization, and reversal of* IL-1β*,* IL-2*, and* IL-6* gene expression. Gene expression and antioxidative enzyme activity were changed by the HBO_2_ and the inflammatory microenvironment in the gut mucosa. Strong correlation of* HIF-1α* mRNA level to* GPx1*,* SOD1*, and* IL-6* mRNA expression suggests involvement of* HIF-1α* in transcriptional regulation of these genes during colonic inflammation and HBO_2_. This is further confirmed by a strong correlation of* HIF-1α* with known target genes VEGF and* PGK1*. Results demonstrate that HBO_2_ has an anti-inflammatory effect in DSS-induced colitis in mice, and this effect is at least partly dependent on expression of* HIF-1α* and antioxidative genes.

## 1. Introduction

Relapsing chronic inflammation found in the gut of individuals affected by inflammatory bowel diseases (IBD) is a result of several overlapping factors, including dysregulation of the immune response to the enteric microbiota, genetic susceptibility, and environmental factors [1, 2]. Reactive oxygen (ROS) and nitrogen (RNS) species generated by inflammatory cells during an immune response create oxidative stress and are considered as important factors contributing to the pathogenesis of IBD. Lymphocytes, neutrophils, and macrophages activated during the gut inflammation produce high amounts of ROS/RNS destroying surrounding tissue [3]. Although oxidative stress is a major factor in the inflamed tissue leading towards necrosis, DNA damage, and carcinogenesis, certain amounts of ROS and other free radicals have an indispensable role in regulating different cell signalling pathways [4]. In addition, some immune cells, namely, the macrophages and neutrophils, use ROS to combat the microorganisms responsible for the infection.

Current therapies of IBD, including immunosuppressive drugs, antibiotics, and biological drugs, are efficient in controlling the course of the disease. However, for many affected individuals, conventional therapy becomes an inadequate choice for long-term treatment because of significant side effects and risk factors, such as increased cancer risk, development of tuberculosis, and heart failure [5–7]. In recent years, hyperbaric oxygen (HBO_2_) therapy has been introduced as a possible additional treatment for IBD patients, especially in the case of refractory disease when the standard therapy is ineffective.

HBO_2_ involves exposure to 100% oxygen under pressure greater than 1 atmosphere of absolute pressure (ATM). It is a well-established procedure frequently applied in the medical practice, especially effective in treating wounds of various aetiologies [8–11]. HBO_2_ increases blood and tissue oxygen saturation resulting also in enhanced production of ROS and RNS [12]. Previous studies have verified that the clinical efficacy of HBO_2_ derives from modulation of intracellular transduction cascades, leading to synthesis of growth factors which promote wound healing, neoangiogenesis, and ameliorates postischemic and postinflammatory injuries [13]. Additional investigations on the mechanism underlying HBO_2_-induced wound healing have revealed a central role of hypoxia inducible factor-1 alpha (*HIF-1α*) as transcriptional regulator of genes involved in angiogenesis, energy metabolism, and cell proliferation [9, 14, 15]. A further important function attributed to the* HIF-1α* is modulation of the immune responses, including the helper T-cell differentiation towards regulatory (Treg) versus Th17 phenotype [16, 17] and its strong anti-inflammatory activity in the gastrointestinal mucosa and hypoxic epithelium as a result of the transactivation of specific genes encoding for barrier-protective elements such as mucins [18–20].

Hypoxia and ROS/RNS can induce stabilization of* HIF-1α* leading to the activation of the hypoxia signal transduction pathway [21]. Increased oxidative stress in the gut mucosa has been verified in humans suffering from ulcerative colitis [22, 23], as well as in experimentally induced colitis in animals [24, 25]. A recent study revealed increased activity of antioxidative enzymes and reduced oxidative stress in the inflamed gut mucosa following HBO_2_ exposure [24]; however, specific mechanisms inducing activation of antioxidative enzymes in the inflamed colonic tissue upon HBO_2_ remain unknown.

Since there is evidence that the intracellular redox status is in a close correlation with the inflammatory microenvironment, and it can also be changed by HBO_2_, the aim of this study was to investigate the effects of HBO_2_ on the mRNA expression of* HIF-1α*, proinflammatory cytokines, and antioxidative enzymes in the gut and peripheral lymphoid organs of BALB/c mice with DSS-induced colitis. An additional aim was to assess the activity of antioxidative enzymes and whether* HIF-1α* gene expression regulation during the gut inflammation and HBO_2_ treatment correlates with the changes in antioxidative and proinflammatory gene expression. Our findings reveal that HBO_2_ treatment may effectively modulate the intestinal milieu in inflammatory conditions involving* HIF-1α*-mediated regulation of antioxidative gene expression.

## 2. Materials and Methods

### 2.1. Animals

BALB/c mice, obtained from Charles River (Calco, Italy), were bred at the animal facility of the Medical Faculty Osijek (Croatia). Mice were provided with standard rodent chow (Mucedola, Settimo Milanese, Italy) and water* ad libitum*. The experimental facility was maintained at 22 ± 2°C, 55 ± 5% humidity, and 12-hour light/dark cycle. All procedures involving live animals were conducted in accordance with the European Guidelines for the Care and Use of Laboratory Animals (directive 86/609/EEC) and were approved by the local Ethical Committee (Faculty of Medicine, University of Osijek) and Croatian Ministry of Agriculture.

### 2.2. Experimental Design

For each experiment male mice at the age of 10–12 weeks were randomized into 4 groups (*n* = 4-5 mice/group/experiment): control mice (CTRL), control mice undergoing HBO_2_ (CTRL + HBO_2_), mice receiving dextran sodium sulphate (DSS), and DSS treated mice undergoing HBO_2_ (DSS + HBO_2_). The average body weight of the mice at the time of inclusion into the study was 22.8 ± 0.4 g. Colitis was induced by 5% (w/v) of DSS (Mr 36.000–50.000, MP Biomedicals, Illkrich, France) in drinking water* ad libitum* for 7 consecutive days [26, 27]. The HBO_2_ treatment was initiated at day 1 and was administered twice a day, 12 hours apart, until the end of experiment (the last session was applied in the morning of day 8; 15 sessions in total). During one HBO_2_ session, mice were exposed to 100% O_2_ for 60 minutes at 2.4 bars with addition of 15 minutes for gradual compression and decompression. Mice were sacrificed by cervical dislocation on day 8, after the morning HBO_2_ session. Disease activity index (DAI) was assessed by daily measurement and scoring of animal body weight loss, stool consistency, and the presence of occult or gross blood per rectum. Measurements were performed at the same time each day until the end of experiment (day 8). DAI was determined as a sum of body weight loss score (0, none; 1, 1–5% loss; 2, 5–10%; 3, 10–15%; 4, >15%), stool consistency score (0, normal; 2, loose stool; 4, diarrhea), and score of occult/gross bleeding (0, normal; 2, occult bleeding; 4, gross hematochezia). Mice were sacrificed by cervical dislocation on day 8 following the last morning HBO_2_ treatment. Colons, mesenteric lymph nodes (MLN), and spleens were collected for further analysis. Colon length was determined for each animal.

### 2.3. Histological Assessment of Colitis

Colonic tissue was removed immediately after the animals were sacrificed, washed in PBS, and fixed in 4% paraformaldehyde. After 72 hours fixed tissue was embedded in paraffin and cut into a series of 6 *μ*m thick sections. Slides were dried, deparaffinised, rehydrated, and stained with haematoxylin and eosin. Histological disease activity was assessed by an experienced histologist, blinded for clinical information. Histological evaluation was preformed according to modified Geboes score as follows [28]:

*Grade 0 (structural (architectural changes)):*

 0: no abnormality,
 1: mild abnormality,
 2: mild or moderate diffuse or multifocal abnormalities,
 3: severe diffuse or multifocal abnormalities.

*Grade 1 (chronic inflammatory infiltrate):*

 0: no increase,
 1: mild but unequivocal increase,
 2: moderate increase,
 3: marked increase.

*Grade 2 (lamina propria leukocytes):*

 0: no increase,
 1: mild but unequivocal increase,
 2: moderate increase,
 3: marked increase.

*Grade 3 (intraepithelial neutrophils):*

 0: none,
 1: <5% crypts involved,
 2: <50% crypts involved,
 3: >50% crypts involved.

*Grade 4 (crypt destruction):*

 0: none,
 1: probable, local excess of neutrophils in part of crypt,
 2: probable, marked attenuation.

*Grade 0 (structural (architectural changes)):*

 3: unequivocal crypt destruction.

*Grade 5 (erosion or ulceration):*

 0: no erosion, ulceration, or granulation tissue,
 1: recovering epithelium + adjacent inflammation,
 2: probable erosion focally stripped,
 3: unequivocal erosion,
 4: ulcer or granulation tissue.

Slides were analysed using light microscopy at magnifications 40x, 100x, 200x, and 400x and photographed at 200x (Olympus® BX50 microscope, Olympus C-5050 digital camera, and QuickPHOTO PRO imaging software (Promicra s.r.o., Prague, Czech Republic)).

### 2.4. Isolation of Lamina Propria Lymphocytes

After isolation, the colon was cleaned of intestinal content and freed of fat tissue, washed in DMEM (Sigma Aldrich, Steinheim, Germany), cut into 5 cm long pieces, and, while shaken at 100 rpm, incubated at 37°C for 20 minutes in DMEM with 25 mM EDTA. The tissue was then thoroughly washed in PBS buffer for at least 5 times, cut into 2 mm long strips, and digested in DMEM containing 5 U/mL DNase (Roche, Mannheim, Germany) and collagenase II (Gibco, Paisley, UK) at 37°C for 20 min. Following this, supernatant was removed, and the previous step was repeated until complete digestion of the tissue was achieved. The supernatant was then filtered through a 100 *μ*m sized filter; DMEM + 2% FBS (Sigma Aldrich, Steinheim, Germany) was added and centrifuged at 800 ×g for 10 min at room temperature. Cells were resuspended in 5 mL of 40% Percoll (GE Healthcare, Uppsala, UK), overlaid on 4 mL of 80% Percoll, and centrifuged at 900 ×g/20 min/4°C. Lymphocytes from the interphase were collected and analysed by flow cytometry.

### 2.5. Flow Cytometry

Lymphocytes were isolated from the mesenteric lymph nodes (MLN) and spleen by teasing apart the organs between the frosted ends of two microscopic slides. The cells were incubated with a mixture of PE anti-CD4 (clone GK1.5, ExBio antibodies), FITC anti-B220 (clone RA3-6B2, obtained from the American Type Culture Collection and conjugated with FITC using standard procedures), and APC anti-CD3 antibodies (clone 145-2C11, ExBio antibodies) and the other panel with PerCP anti-CD45 (clone 30-F11, BD Biosciences), FITC anti-Gr-1 (clone RB6-8C5, BD Biosciences), and PE anti-F4/80 (clone BM8, StemCell Technologies Inc.) or PE anti-CD4 (clone MEM-241, ExBio antibodies) and PerCP anti-CD8 (clone MEM-31, ExBio antibodies) antibodies. Dead cells were excluded based on 7-aminoactinomycin D (7-AAD) (Applichem, Darmstadt, Germany) staining. At least 20,000 live cells were collected by a BD FACS Canto II cytometer (FACS Canto II, Becton Dickinson, San Jose, CA, USA) and analysed using the FlowLogic software (Inivai Technologies, Mentone, Australia).

### 2.6. Measurement of Intracellular ROS Level

To assess hydrogen peroxide (H_2_O_2_) and peroxynitrite (ONOO^−^) level, 10^6^ lymphocytes isolated from MLN and spleens were incubated for 30 min on +4°C with 10 *μ*M dichlorofluorescein diacetate (DCF-DA) (Biomol, Hamburg, Germany), washed for 5 min at 400 ×g on +4°C, and analysed with the FACS Canto II. Following this, cells were stimulated with 100 nM phorbol 12-myristate 13-acetate (PMA, Calbiochem, Darmstadt, Germany), incubated for 30 min, and analysed for the second time. At each measurement minimum of 10,000 target cells were analysed. Data are expressed as the median fluorescence intensity (MFI) ± s.e.m.

### 2.7. Real-Time PCR

Colon, MLN, and spleen samples were isolated, snap frozen in liquid nitrogen, and stored at −80°C till analysis. Total RNA was extracted using ONE STEP RNA Reagent (BIO BASIC Inc., Markham, Ontario, Canada) according to manufacturer's protocol. RNA purity and concentration was assessed by NanoPhotometer® P-Class P330-30 (Implen, Munich, Germany). In order to purify RNA from all polysaccharides, including DSS, an additional purification step using 8 M LiCl was performed [29], followed by the standard genomic DNA purification step using Deoxyribonuclease I kit (Sigma Aldrich, St Louis, MO, USA). One microgram of RNA was used for cDNA synthesis by High Capacity cDNA kit with RNase Inhibitor (Applied Biosystems, Foster City, CA, USA). Real-time PCR was performed on CFX96 system (Bio Rad, Singapore) to assess relative expression of catalase (*CAT*), glutathione peroxidase 1 (*GPx1*), superoxide dismutase 1 (*SOD1*),* HIF-1α*,* IL-1β, IL-2,* and* IL-6*. Gene expression was normalized to* HPRT1* gene. Primers list is given in the supplementary data (Table 1). Except for the primers for* IL-6* gene published by Jeong et al. [30], all other primers were custom made using Primer 3 software. Messenger RNA expression was determined using SsoFast EvaGreen Supermix (Bio Rad, Singapore). Data are presented as mean ± s.e.m.

### 2.8. Antioxidative Enzymes Activity Measurement

Frozen tissue samples were homogenized with liquid nitrogen and weighed. Tissue powder was additionally homogenized in 100 mM phosphate buffer solution (pH 7.0) containing 1 mM EDTA (1 : 10, w/v) using Ultra turrax T10 homogenizer (IKA, Staufen, Germany) while kept on ice. Tissue homogenates were sonicated for 30 seconds on ice in three 10 seconds intervals and then centrifuged at 20,000 ×g for 15 minutes at 4°C. Supernatant was collected, aliquoted, and stored at −80°C till analysis. CAT, GPx, and SOD enzyme activities were determined using a Lambda 25 UV-Vis spectrophotometer equipped with UV WinLab 6.0 software package (Perkin Elmer for the Better, Massachusetts, USA).

Catalase (EC 1.11.1.6) activity was estimated spectrophotometrically using H_2_O_2_ as a substrate [31]. The reaction mixture consisted of 10 mM H_2_O_2_ in 50 mM phosphate buffer pH 7.0. Changes in absorbance of the reaction mixture were measured at 240 nm during 2 minutes after the sample addition. One unit of activity corresponds to the loss of 1 *μ*mol of H_2_O_2_ per minute. CAT activity was calculated using molar extinction coefficient (*ε* = 0.04 mM cm^−1^) and expressed as U mg^−1^ protein.

To assess glutathione peroxidase (EC 1.11.1.9) activity a modified method described by Wendel [32] using H_2_O_2_ as a substrate was employed. GPx activity was determined indirectly by measuring the rate of NADPH oxidation to NADP+, accompanied by a decrease in absorbance at 340 nm. The assay mixture consisted of 50 mM phosphate buffer with 0.4 mM EDTA and 1 mM sodium azide (pH 7.0), 0.12 mM NADPH, 3.2 units of GR, 1 mM glutathione, and 0.0007% (w/w) H_2_O_2_ in a total volume of 1.55 mL. One unit catalyses the oxidation by H_2_O_2_ of 1.0 *μ*mole of reduced glutathione to oxidized glutathione per minute at pH 7.0 and 25°C. GPx activity was calculated using molar extinction coefficient for NADPH (*ε* = 6.220 mM cm^−1^) and expressed as U mg^−1^ protein.

Superoxide dismutase (EC 1.15.1.1.) activity was determined using cytochrome C (0.05 mM) as an inhibitory molecule in PBS buffer saline with 0.1 mM EDTA in system xanthine (1 mM)/xanthine oxidase (50 U) by Flohe method [33].

Total soluble protein concentration in protein extracts was determined by Bradford reagent (Sigma Aldrich, Steinheim, Germany) following manufacturer's protocol and using bovine serum albumin as a standard.

### 2.9. Statistical Analysis

Normal distribution was assessed by the Shapiro Wilk test. Within groups differences were tested by one-way ANOVA or Kruskal-Wallis test followed by the Holm-Sidak/Tukey or Dunn's* post hoc* multiple comparison procedure, respectively (Sigma plot 11.0, SigmaStat Inc., San Jose, CA, USA). In some cases, the Student *t*-test and Mann-Whitney* U* Statistic were used to compare the differences between the two groups in the case of normally distributed variables and variables that violated assumption of normality, respectively. Spearman's correlations were calculated where appropriate (Sigma plot 11.0, SigmaStat Inc.). DAI results were analysed by two-way ANOVA and Bonferroni* post hoc* test (GraphPad Prism 5.0, GraphPad Software, Inc., La Jolla, CA, USA). A* P* value of < 0.05 was considered statistically significant for all procedures. All data are presented as mean values ± standard error of mean (s.e.m.).

## 3. Results

### 3.1. HBO_2_ Ameliorates the Course of DSS-Induced Colitis

**Figure 1:**
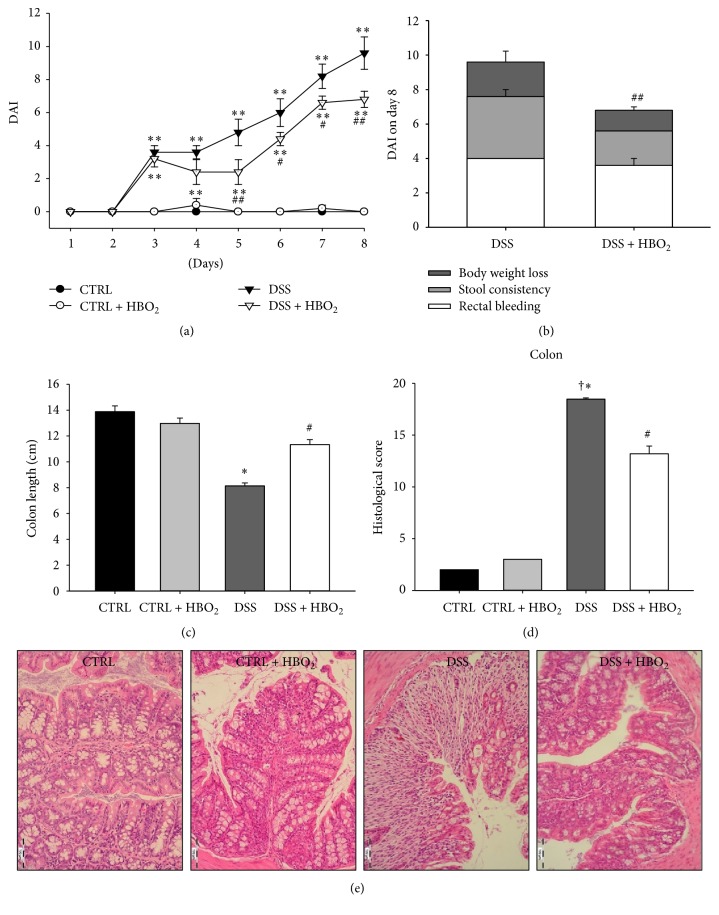
HBO_2_ ameliorates the course of DSS-induced colitis in BALB/c mice. Male BALB/c mice at the age of 10–12 weeks were randomly assigned into 4 groups (*n* = 5 mice/group/experiment): CTRL: control mice, CTRL + HBO_2_: control mice undergoing HBO_2_ (60 min/2.4 ATM, 2x/day, days 1–8), DSS: mice receiving dextran sodium sulphate (5% w/v, days 1–7), and DSS + HBO_2_: DSS treated mice undergoing HBO_2_. (a) Disease activity index (DAI) assessed by daily scoring of the body weight change, stool consistency, and occult/gross rectal bleeding; (b) the stacked bars on the bar chart represent average score of each symptom in the total DAI score for particular experimental group, including weight changes, stool consistency, and rectal bleeding score; (c) colon length measured in cm; (d) histological samples of gut tissue were stained with haematoxylin and eosin and severity of colitis assessed using modified Geboes score; (e) representative histological samples of distal colon, CTRL and CTRL + HBO_2_: normal intact colonic mucosa, DSS: the mucosa shows severe inflammation and ulceration extending into the deep portions of the mucosa with loss of crypts, and DSS + HBO_2_: structural restoration with mild inflammatory infiltration and crypt distortion; magnification 200x. Presented data (mean ± s.e.m.) are representative results from one experiment with *n* = min. 5 mice/group. ^∗^Statistically different from CTRL *P* < 0.05 and ^*∗∗*^ *P* < 0.01; ^#^statistically different from DSS *P* < 0.05 and ^##^ *P* < 0.01; ^†^statistically different from CTRL + HBO_2_.

In order to determine the effects of HBO_2_ on the course of acute colitis, BALB/c mice were exposed to 5% DSS in the drinking water* ad libitum* and daily monitored for body weight, stool consistency, and occult/gross rectal bleeding to calculate DAI. In this study the DSS treatment induced substantial weight loss, rectal bleeding, loose stool, and colon shortening resulting in significantly higher DAI compared to the control (CTRL) group, starting from day 3 until the end of the experiment (*P* < 0.01; Figure 1(a)). Mice that received DSS and underwent HBO_2_ treatment (DSS + HBO_2_ group) also presented with significantly higher DAI compared to the CTRL group; however, in this group of mice HBO_2_ treatment significantly reduced DAI compared to the DSS mice, starting from day 5 throughout day 8 (days 5 and 8 *P* < 0.01; days 6 and 7 *P* < 0.05; Figure 1(a)). In addition, average colon length in the DSS group of mice was significantly shorter (8.14 ± 0.23 cm) compared to the CTRL group (13.88 ± 0.45 cm; *P* < 0.0001), while this effect was significantly ameliorated by HBO_2_ treatment in the DSS + HBO_2_ group (11.34 ± 0.38 cm) compared to the DSS group (*P* = 0.0001, Figure 1(b)). DSS induced colitis resulted in significant body mass loss compared to CTRL group, irrespective of HBO_2_ treatment (11.89 ± 0.03% and 8.24 ± 0.01% in the DSS and DSS + HBO_2_ group, resp.; *P* < 0.001). Mice undergoing HBO_2_ presented with reduced body mass loss. CTRL and CTRL + HBO_2_ gained body mass during the experiment, 8.25 ± 0.2% and 0.33 ± 0.01%, respectively.

Histological assessment of the colon revealed severe inflammation and ulceration extending into the deep portions of the mucosa with loss of crypts and with increased number of lamina propria leukocytes in DSS group of mice. We also found structural changes of mucosa in the colonic tissue of DSS + HBO_2_ mice but with reduced infiltration of inflammatory cells and decreased crypt distortion. When compared to the DSS + HBO_2_ group, the DSS group had significantly higher total histological score as well as individual scores (see modified Geboes score in Section 2.3, Figure 1(d), *P* < 0.001), except for the intraepithelial neutrophil infiltration score (*P* = 0.104).

**Figure 2:**
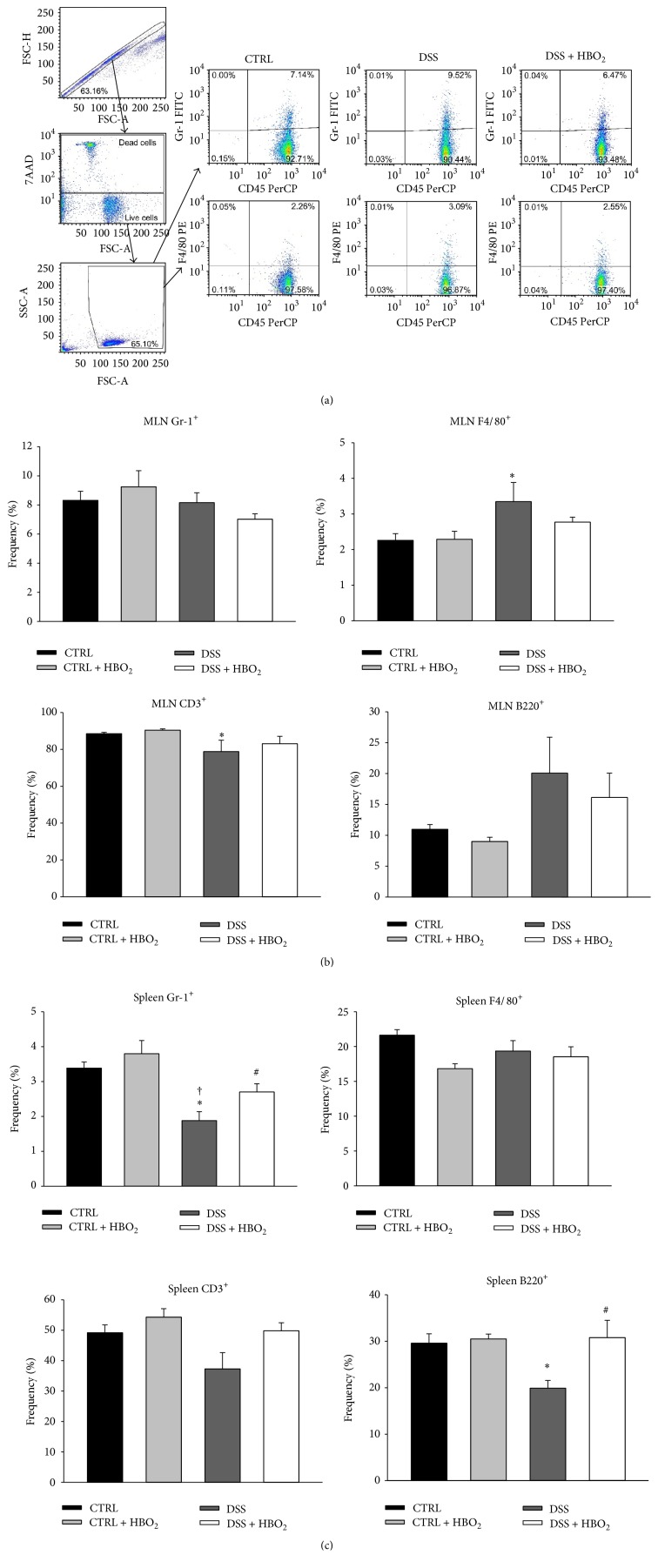
HBO_2_ changes immune cell frequencies in the mesenteric lymph nodes (MLN) and the spleen of BALB/c mice with DSS induced colitis. BALB/c mice at the age of 10–12 weeks were randomly assigned into 4 groups (*n* = min. 5 mice/group/experiment): CTRL: control mice, CTRL + HBO_2_: control mice undergoing HBO_2_ (60 min/2.4 ATM, 2x/day, days 1–8), DSS: mice receiving dextran sodium sulphate (DSS, 5% w/v, days 1–7), and DSS + HBO_2_: DSS treated mice undergoing HBO_2_. Representative dot plots of MLN obtained by flow cytometry, illustrating the gating strategy for CD45^+^ Gr-1^+^ and CD45^+^ F4/80^+^ cells in MLN of CTRL, DSS, and DSS + HBO_2_ groups. Doublets were excluded by forward scatter area (FSC-A) versus forward scatter height (FSC-H) and the dead cells using 7-AAD. (a) Frequency of CD45^+^ Gr-1^+^cells, CD45^+^ F4/80^+^ cells, CD3^+^ T cells, and B220^+^ B cells in MLN (b) and spleen (c). Data are presented as mean ± s.e.m.% of single, live leukocytes (for Gr-1^+^ and F4/80^+^ cells) or single, live lymphocytes (for CD3^+^ and B220^+^ cells). ^∗^Statistically different from CTRL, *P* < 0.05; ^†^statistically different from CTRL + HBO_2_, *P* < 0.05; ^#^statistically different from DSS, *P* < 0.05. 7-AAD, 7-aminoactinomycin D.

Distribution of inflammatory cells among the peripheral lymphoid organs, including Gr-1^+^ leukocytes (monocytes and neutrophils), F4/80^+^ leukocytes (monocytes), CD3^+^ T lymphocytes, and B220^+^ B lymphocytes (Figure 2), was assessed at the end of the experiment. In the MLN, frequencies of Gr-1^+^ cells did not differ among the experimental groups, while DSS induced a significant increase in F4/80^+^ and decrease in CD3^+^ cell frequencies (*P* < 0.05 and *P* = 0.032, resp.; Figure 2(b)). These findings in the DSS group were accompanied by a B-cell increase which was not statistically significant. HBO_2_ alone had no effect on the cell frequencies in MLN of control mice, whereas it substantially ameliorated these changes in mice with DSS-induced colitis (DSS + HBO_2_ group) but without reaching statistical significance.

In the spleen, Gr-1^+^ cell frequencies were significantly decreased in the DSS group compared to CTRL (*P* = 0.006) and CTRL + HBO_2_ groups (*P* < 0.001; Figure 2(a)). HBO_2_ treatment reversed Gr-1^+^ cell frequencies to control values in the DSS + HBO_2_ group (*P* = 0.056; Figure 2(a)). In addition, the DSS mice showed reduced frequencies of B220^+^ lymphocytes compared to the CTRL group (*P* = 0.016), and HBO_2_ treatment abolished these effects in the DSS + HBO_2_ group (*P* = 0.034; Figure 2(c)). In addition, our study revealed that DSS-induced immune responses in the MLN and the spleen were significantly dampened by HBO_2_ treatment.

**Figure 3:**
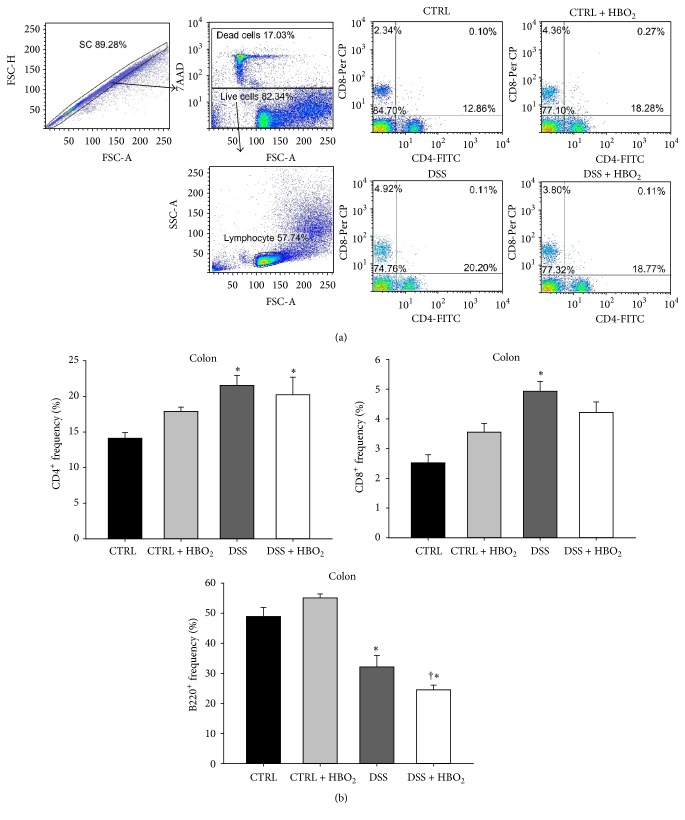
HBO_2_ induced changes in CD4^+^ and CD8^+^ T cell frequencies in the colon of BALB/c mice with DSS induced colitis. BALB/c mice at the age of 10–12 weeks were randomly assigned into 4 groups (*n* = min. 4 mice/group/experiment): CTRL: control mice, CTRL + HBO_2_: control mice undergoing HBO_2_ (60 min/2.4 ATM, 2x/day, days 1–8), DSS: mice receiving dextran sodium sulphate (DSS, 5% w/v, days 1–7), and DSS + HBO_2_: DSS treated mice undergoing HBO_2_. (a) Representative dot plots of colon lamina propria lymphocyte obtained by flow cytometry, illustrating the gating strategy for CD4^+^ and CD8^+^ lymphocytes, and (b) measured frequencies of colon CD4^+^ and CD8^+^ lymphocytes. Doublets were excluded by forward scatter area (FSC-A) versus forward scatter height (FSC-H) and the dead cells using 7-AAD. Data are presented as mean ± s.e.m.% of single, live lymphocytes. ^∗^Statistically different from CTRL, *P* < 0.05; ^†^statistically different from CTRL + HBO_2_.

Frequency of CD4^+^ cells among the colon lamina propria lymphocytes of DSS and DSS + HBO_2_ groups was significantly increased compared to the CTRL group (*P* = 0.015 and *P* = 0.047, resp.), while the frequency of CD8^+^ cells was significantly increased only in the DSS group when compared to the CTRL group (*P* = 0.011; Figure 3).

### 3.2. Inflammation of Colonic Mucosa and HBO_2_ Treatment Induce Changes in Antioxidative Enzymes Gene Expression and* HIF-1α* Gene Regulation

**Figure 4:**
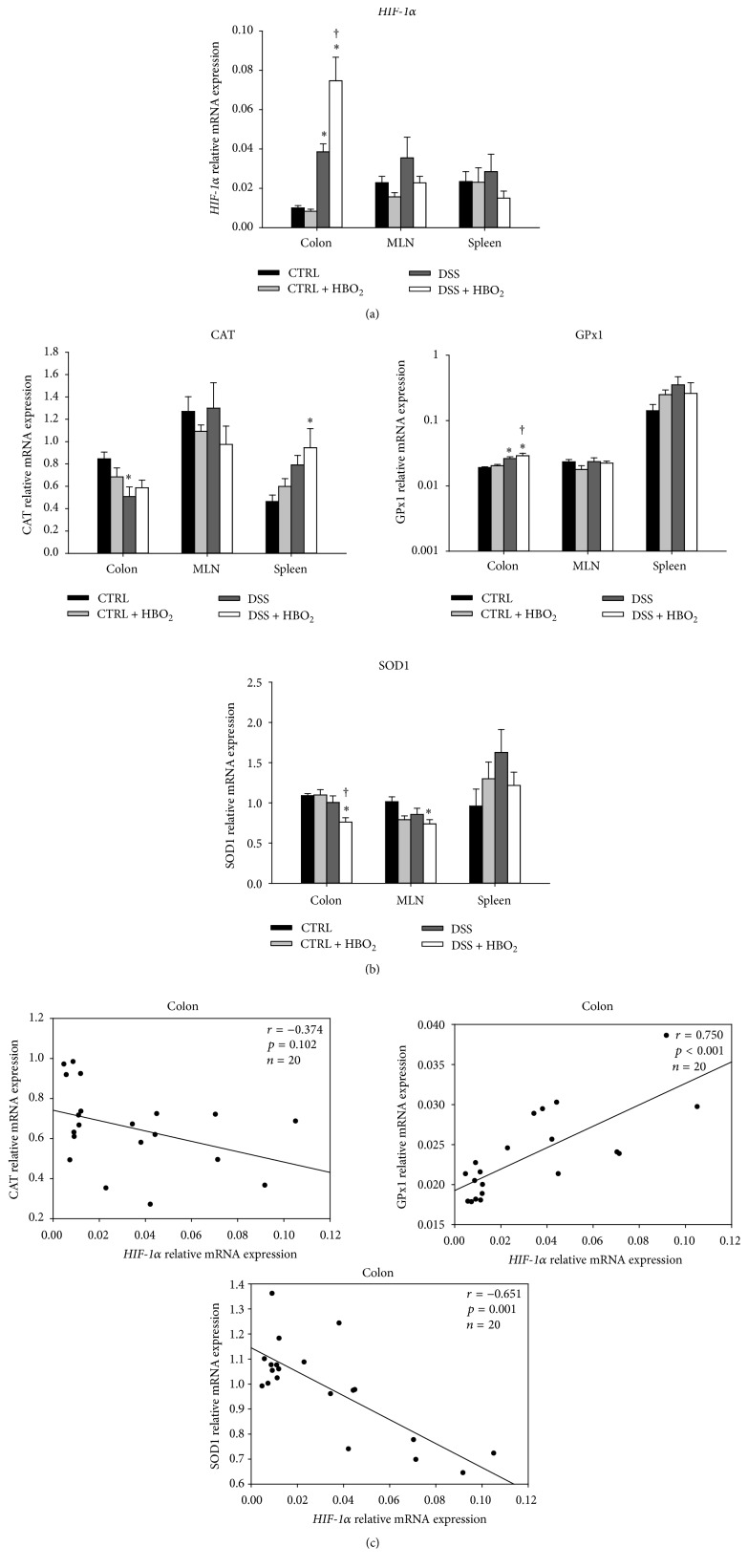
Relative mRNA expression of* HIF-1α*,* CAT*,* GPx1,* and* SOD1* in colon, MLN, and spleen (a, b) and their correlation to the* HIF-1α* gene expression in colonic tissue (c). Relative mRNA expression was measured by real-time PCR; all measured genes were normalized to the* HPRT1* gene expression. BALB/c mice at the age of 10–12 weeks were randomly assigned into 4 groups (*n* = min. 5/group/experiments): CTRL: control mice, CTRL + HBO_2_: control mice undergoing HBO_2_ (60 min/2.4 ATM, 2x/day, days 1–8), DSS: mice receiving dextran sodium sulphate (DSS, 5% w/v, days 1–7), and DSS + HBO_2_: DSS treated mice undergoing HBO_2_. Data are presented as mean ± s.e.m. of two independent experiments, each with min. 5 mice/group. ^∗^Statistically different from CTRL, *P* < 0.05; ^†^statistically different from CTRL + HBO_2_, *P* < 0.05.

Inflammatory conditions have been known to include the enhanced production of ROS and other oxidative mediators that may affect transcriptional regulation via* HIF-1α*. To investigate the role of* HIF-1α* in the regulation of the antioxidative response/capacity during DSS-induced colitis and HBO_2_ treatment,* HIF-1α, CAT*,* GPx1*, and* SOD1* mRNA expressions were determined using quantitative PCR method.* HIF-1α* mRNA expression was significantly changed by the HBO_2_ treatment and the inflammatory microenvironment in the gut mucosa. DSS-induced colitis resulted in significant upregulation of* HIF-1α* gene in colonic mucosa (*P* = 0.008 for DSS group compared to CTRL), and the HBO_2_ treatment further increased* HIF-1α* mRNA expression in the DSS + HBO_2_ group (*P* = 0.028 compared to CTRL; Figure 3(a)). In addition, the activity of* HIF-1α* protein was indirectly confirmed by measuring mRNA expression of well-established* HIF-1α* target genes,* VEGF* and* PGK1*. Both genes showed strong positive correlation to the* HIF-1α* mRNA (Supplementary Figure 1) (see Supplementary Material available online at http://dx.doi.org/10.1155/2016/7141430). There was also a tendency for upregulation of* HIF-1α* gene in MLN and spleens of the DSS group and its downregulation via HBO_2_ in the DSS + HBO_2_ group (Figure 4(a)); however, these changes did not reach statistical significance.

Inflammation during DSS-induced colitis and the HBO_2_ treatment also induced significant changes in mRNA expression of target antioxidative genes. DSS-treated mice presented with significant downregulation of the* CAT* gene in the colon compared to the CTRL group (*P* = 0.031), while there was a significant upregulation of* CAT* gene in the spleen of the DSS + HBO_2_ mice compared to the CTRL group (*P* = 0.026; Figure 4(b)). In the colon,* GPx1* mRNA expression was increased in the DSS (*P* = 0.034) and the DSS + HBO_2_ (*P* = 0.003) group compared to CTRL group. The upregulation was even greater in mice with DSS-induced colitis that underwent the HBO_2_ treatment (DSS + HBO_2_ group; Figure 4(b)).* SOD1* mRNA expression was significantly reduced in the colon of the DSS + HBO_2_ group compared to CTRL (*P* = 0.008) and CTRL + HBO_2_ (*P* = 0.007) groups. Similar changes in* SOD1* gene expression were also found in the MLN of the DSS + HBO_2_ group (*P* = 0.025 compared to CTRL; Figure 4(b)). To summarize, colitis resulted in* GPx1* gene upregulation and* CAT* gene downregulation, while HBO_2_ downregulated* SOD1* and further upregulated* GPx1* in a tissue-specific manner.

To examine the possible role of* HIF-1α* in transcriptional control of antioxidative genes in the colon, Spearman correlations were calculated. The results revealed a strong negative correlation between* HIF-1α* and* SOD1* (*r* = −0.651, *P* = 0.001) and a positive correlation of* HIF-1α* to the* GPx1* gene (*r* = 0.750, *P* < 0.001), while there was no significant correlation between the* HIF-1α* and the* CAT* gene in the colonic tissue (Figure 4(c)).

### 3.3. HBO_2_ Treatment Reduces Expression of Proinflammatory Genes Upregulated during DSS-Induced Colitis

**Figure 5:**
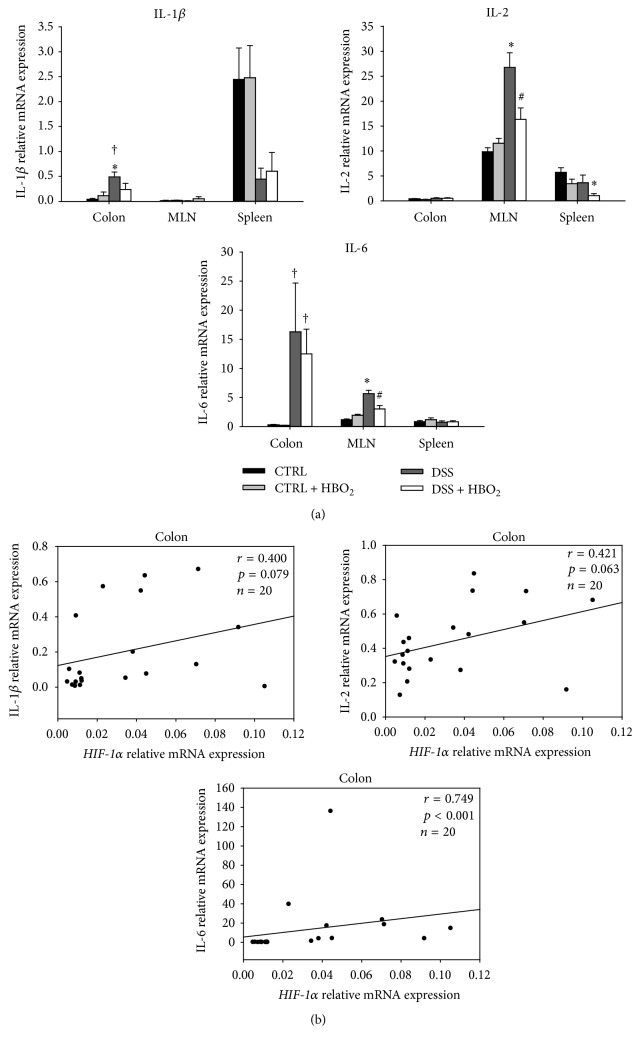
Relative mRNA expression of* IL-1β*,* IL-2,* and* IL-6* in the colon, MLN, and spleen (a) and their correlation to* HIF-1α* gene expression in colonic tissue (b). Relative mRNA expression was measured by real-time PCR; all measured genes were normalized to expression of* HPRT1* gene. BALB/c mice at the age of 10–12 weeks were randomly assigned into 4 groups (*n* = min. 5/group/experiment): CTRL: control mice, CTRL + HBO_2_: control mice undergoing HBO_2_ (60 min/2.4 ATM, 2x/day, days 1–8), DSS: mice receiving dextran sodium sulphate (DSS, 5% w/v, days 1–7), and DSS + HBO_2_: DSS treated mice undergoing HBO_2_. Data are presented as mean ± s.e.m.; ^∗^statistically different from CTRL, *P* < 0.05; ^#^statistically different from DSS, *P* < 0.05; ^†^statistically different from CTRL + HBO_2_, *P* < 0.05.

The early phase of inflammation is mediated by several proinflammatory mediators, which prompted us to assess how HBO_2_ treatment affects their production. We found that gut mucosa inflammation was accompanied with a significant increase in* IL-1β* and* IL-6* gene expression. In the case of* IL-6* gene, this was significant for DSS and DSS + HBO_2_ groups compared to the CTRL + HBO_2_ group (*P* = 0.024 and *P* = 0.021, resp.; Figure 5(a)) in the colon. Furthermore,* IL-6* gene was significantly upregulated in the MLN of the DSS group (*P* = 0.001 compared to CTRL), and HBO_2_ reduced its expression almost to control values in the DSS + HBO_2_ group (*P* = 0.016 compared to DSS; Figure 5(a)).* IL-1β* mRNA expression in the colonic mucosa was significantly increased during inflammation in DSS group compared to CTRL (*P* = 0.014) and CTRL + HBO_2_ groups (*P* = 0.041).* IL-1β* mRNA levels in the colon of the DSS + HBO_2_ group did not significantly differ from the control groups, suggesting that HBO_2_ treatment blocked the increase of* IL-1β* gene expression in the inflamed mucosa.

*IL-2* gene was significantly upregulated in the MLN of the DSS group compared to the CTRL group (*P* = 0.003), and HBO_2_ treatment resulted in its significant downregulation in the DSS + HBO_2_ group (*P* = 0.032). Similarly,* IL-2* gene was significantly downregulated in the spleen of mice from the DSS + HBO_2_ group compared to the CTRL group (*P* = 0.025). In addition, there was a strong positive correlation between* HIF-1α* and* IL-6* gene (*r* = 0.749, *P* < 0.001; Figure 5(b)), while there was no correlation between* HIF-1α* and* IL-1β* or* IL-2* genes in the colonic tissue (Figure 5(b)).

### 3.4. Colonic Inflammation and HBO_2_ Treatment Induce Changes in the Activity of Antioxidative Enzymes

In addition to their mRNA expression, we also tested the enzymatic activity of antioxidative enzymes. We found that both the inflammation and the HBO_2_ treatment* per se* were able to change the activity of antioxidative enzymes in the colonic mucosa and the peripheral lymphoid organs (MLN and spleen). In spleen HBO_2_ treatment* per se* induced significant increase of SOD activity (*P* = 0.040 compared to CTRL). Mice with DSS-induced colitis presented with significantly increased activity of SOD (*P* = 0.012 compared to CTRL) in the colon and reduced CAT activity in MLN and spleen (*P* = 0.023 and *P* = 0.032, resp.) compared to CTRL group. HBO_2_ treatment did not change SOD activity in the inflamed colonic mucosa, which was comparable to the levels found in DSS and CTRL + HBO_2_ groups (significantly increased compared to CTRL, *P* = 0.010). On the other hand, HBO_2_ treatment significantly increased CAT (*P* = 0.020) and GPx (*P* = 0.001) activities in the spleens of the DSS + HBO_2_.

### 3.5. Lymphocyte H_2_O_2_ and ONOO^−^ Production Remains Unchanged during DSS-Induced Colitis and HBO_2_ Treatment

**Figure 6:**
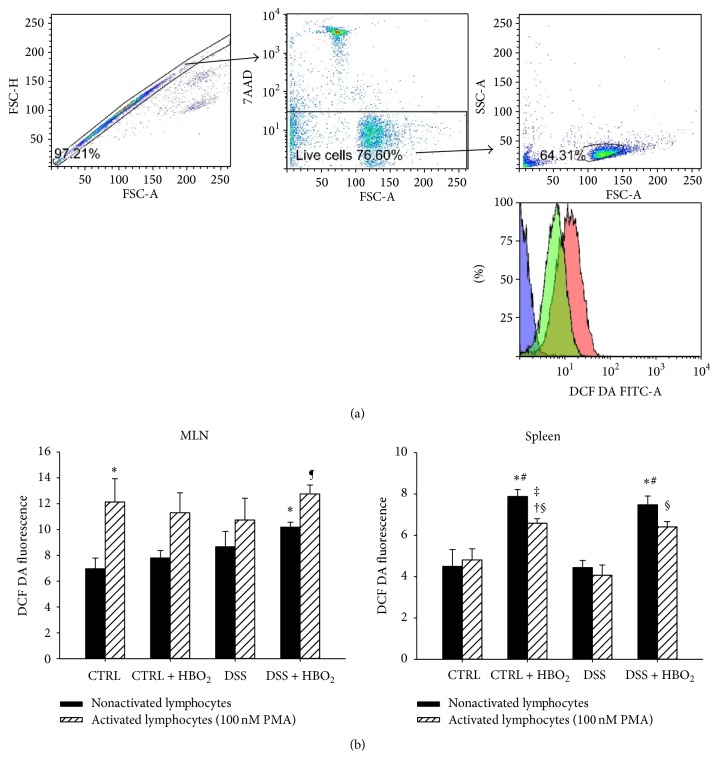
Lymphocyte H_2_O_2_ and ONOO^−^ production during colitis and HBO_2_ treatment. BALB/c mice at the age of 10–12 weeks were randomly assigned into 4 groups (*n* = min. 4 mice/group/experiment): CTRL: control mice, CTRL + HBO_2_: control mice undergoing HBO_2_ (60 min/2.4 ATM, 2x/day, days 1–8), DSS: mice receiving dextran sodium sulphate (DSS, 5% w/v, days 1–7), and DSS + HBO_2_: DSS treated mice undergoing HBO_2_. To assess the basal intracellular H_2_O_2_ and ONOO^−^ levels, lymphocytes isolated from MLN and spleens were stained with 10 *μ*M DCF DA and analysed by flow cytometry (black bars, (b)). Next, lymphocytes were activated with 100 nM PMA for 30 min and the ROS production measurement repeated (lined white bars, (b)). (a) shows a representative lymphocyte gating strategy panel and a histogram of DCF-DA signal from the negative control (blue), resting lymphocytes (green), and the PMA activated lymphocytes (red). Doublets were excluded by forward scatter area (FSC-A) versus forward scatter height (FSC-H) and the dead cells by 7-AAD staining. Data are presented as mean ± s.e.m.; ^∗^statistically different compared to CTRL, *P* < 0.05; ^#^statistically different compared to DSS group, *P* < 0.05; ^†^statistically different from CTRL + HBO_2_, *P* < 0.05; ^‡^statistically different compared to activated lymphocytes of CTRL group, *P* < 0.05; ^§^statistically different from the activated lymphocytes of DSS group, *P* < 0.05. DCF DA: dichlorofluorescein diacetate; PMA: phorbol myristate acetate; 7-AAD: 7-aminoactinomycin D; ^¶^statistically different from nonactivated lymphocytes DSS + HBO_2_.

Immune cells at the site of inflammation and in the peripheral lymphoid organs are an important source of ROS [3]. Therefore we assessed the basal levels of intracellular H_2_O_2_ and ONOO^−^ and their production upon PMA-induced activation in the lymphocytes isolated from MLN and spleens of the mice from all experimental groups (Figure 6). Basal H_2_O_2_ and ONOO^−^ production in the MLN was not significantly different among the groups, except for the lymphocytes from the DSS + HBO_2_ group which presented with a significant increase of H_2_O_2_ and ONOO^−^ levels compared to the CTRL group (*P* = 0.033). PMA stimulation resulted in increased intracellular H_2_O_2_ and ONOO^−^ production, although statistically significant only for CTRL (*P* = 0.031) and DSS + HBO_2_ (*P* = 0.012) groups.

In the spleen, HBO_2_ increased lymphocyte H_2_O_2_ and ONOO^−^ production in CTRL + HBO_2_ and DSS + HBO_2_ groups (*P* = 0.004 and *P* = 0.007 compared to the CTRL; and *P* = 0.005 and *P* = 0.009 compared to the DSS group). Their production after PMA-induced activation was decreased in all experimental groups except the CTRL group; however, this effect reached statistical significance only in the CTRL + HBO_2_ group (*P* = 0.018 compared to unstimulated lymphocytes).

## 4. Discussion

In the present study, the experimental model of DSS-induced colitis in BALB/c mice was employed to explore the effects of HBO_2_ on the antioxidative enzymes, transcription factor* HIF-1α,* and proinflammatory cytokine genes during colonic inflammation and their role in modulating the course of the disease via HBO_2_ treatment. The most important findings are that (a) HBO_2_ significantly reduces symptoms and severity of DSS-induced colitis, as evidenced by clinical appearance, contraction of the immune cell expansion and mobilization, and reversal of* IL-1β, IL-2,* and* IL-6* gene expression; (b) HBO_2_ modulates the expression of antioxidative enzyme genes and enzyme activities during colitis; and (c) HBO_2_ enhances* HIF-1α* mRNA expression in the inflamed colonic tissue which is in a strong correlation with* GPx1, SOD1,* and* IL-6* mRNA expression.

Several previous studies in animals and humans demonstrated the positive effects of HBO_2_ treatment in influencing the severity of colitis and reducing gut mucosa inflammation [27, 34]; however, data on the precise underlying mechanisms are scarce. Considerably more data on the beneficial anti-inflammatory effects of HBO_2_ are available for other conditions such as septic shock, ischemia/reperfusion injuries, and atherogenesis, where the previous studies reported reduced proinflammatory cytokine expression, suppressed development of Th cells, shrinking of spleen and lymph nodes, decreased responses to antigens, and reduced frequencies of circulating leukocytes [35–42]. Although this is the first animal study investigating the effects of HBO_2_ performed on DSS-induced colitis in BALB/c mice and correlating it with the immune cell frequencies, our results are in line with previous findings on the changes associated with DSS-induced acute immune response, as well as on the effects of HBO_2_ on the antioxidative enzyme activities determined in other animal models, such as TNBS and acetic acid induced colitis in rats [27, 43, 44]. During colitis mice presented with decreased T and B cell frequencies in the spleen and reduced T cell frequencies in the MLN, suggesting that lymphocytes are recruited from the peripheral lymphoid organs and probably migrate to the inflamed colonic mucosa. One element of the beneficial effect of HBO_2_ may be linked to normalized T and B cells frequency in the MLN and spleen of mice with DSS-induced colitis after hyperbaric treatment (Figures 1 and 2). By measuring CD4 and CD8 lymphocyte in colon we confirm our hypothesis of T-cell recruitment from the peripheral lymphoid organs and their migration to the inflamed colonic mucosa, as well as immunomodulatory effect of HBO_2_. In our model HBO_2_ did not affect cell frequencies in the peripheral lymphoid organs of control mice, in contrast to previous findings where HBO_2_ treatment* per se* was able to change lymphocyte subset populations in the spleen [45]. The observed differences may be due to different oxygen tension applied in our study.

For a long time macrophages and neutrophils have been considered as immune cells exclusively producing proinflammatory cytokines, chemokines, and large amounts of ROS/RNS contributing to aggravated inflammation. We have found decreased spleen Gr-1^+^ cell frequencies during colitis and their normalization upon HBO_2_ treatment (Figure 2). In addition, we showed increased MLN frequencies of F4/80^+^ cells in DSS group, while HBO_2_ treatment reversed their frequencies almost to control values. These findings indicate that HBO_2_ can modulate distribution of phagocytes by retaining neutrophils in the spleen and instigating macrophage migration towards the site of inflammation, in agreement with previous findings describing inhibited neutrophil infiltration into the gut of mice with DSS-induced colitis [46]. Furthermore, HBO_2_ treatment alone did not change the expression of proinflammatory cytokines in the colon, MLN, or spleen of the control mice; however, DSS-induced colitis resulted in a significant* IL-1β* and* IL-6* gene upregulation in the colonic tissue and* IL-2* gene upregulation in the MLN (Figure 5). Consistent with previous studies, HBO_2_ treatment abolished these effects, further confirming its anti-inflammatory potential [47–49].

Several animal studies on the effects of HBO_2_ on the experimental colitis reported an increased antioxidative capacity and changes in antioxidative enzyme activity [24, 25]. It has been proposed that an optimal HBO_2_ treatment could generate ROS which would function primarily as intermediates in the antioxidative signalling pathways leading to increased expression of antioxidative enzymes, reduced inflammation, and ameliorated colitis symptoms but would not further damage the colonic tissue [50, 51]. Drenjancevic et al. showed that 24 hours after a two-hour HBO_2_ treatment at 2 bars in rats oxidative stress is not elevated, as evidenced by assessing ferric reducing antioxidant power ability of plasma (FRAP) and thiobarbituric acid reactive substances (TBARS) level [12]. In addition, a recent study also suggests that ROS produced by NADPH oxidase complex are important mediators inducing anti-inflammatory response in autoimmune diseases [52]. Data on the* CAT* mRNA level during DSS-induced colitis and upon HBO_2_ treatment were not available prior to this study. We found that* CAT* mRNA expression is tissue and treatment specific (Figure 4). Colitis resulted in a significant downregulation of* CAT* mRNA expression in the colonic mucosa, and the HBO_2_ treatment induced its upregulation in the spleen of DSS + HBO_2_ group of mice. These results were largely in accordance with our finding on enzymatic catalase activity that was decreased in all measured tissues in the DSS group and reversed to control values in the spleen of DSS + HBO_2_ group (Table 2), as well as with a previous study demonstrating decreased catalase activity in colonic tissue upon DSS treatment [53]. This is also in line with a study on skin transplanted BALB/c mice where HBO_2_ treatment increased catalase, GPx, and SOD activity in the spleen [54]. Furthermore, upregulation of protein and mRNA catalase levels 14 days after HBO_2_ treatment, but not after 7 days, was also observed in the ulcer tissue of patients with diabetic foot, indicating a time-course for the effect of HBO_2_ to prevail [55].

We found that* GPx1* mRNA level was upregulated in the colon of DSS treated mice, irrespective of the HBO_2_ treatment, and there were no significant differences in the* GPx1* mRNA expression in MLN and spleen. In contrast to our findings on mRNA expression, colon GPx enzyme activity was slightly reduced in DSS + HBO_2_ group compared to other groups, which is consistent with previous results obtained in acetic acid induced colitis in rats receiving combined HBO_2_ and ozone treatment [56]. However, other reports indicate decreased GPx and SOD activity in the inflamed distal colon mucosa and the plasma of rats with acetic acid induced colitis, and HBO_2_ normalized GPx but not SOD activity in the colon [24]. The observed discrepancies in the results may be related to the differences in experimental models used among the studies. In addition, in our study HBO_2_ treatment induced enhanced GPx and SOD activity in the spleen of DSS mice which is in contrast to reduced* SOD1* mRNA expression and might be explained by additional SOD2 and SOD3 function in regulation of antioxidative capacity. Although intracellular H_2_O_2_ and ONOO^−^ levels were slightly increased during inflammation and HBO_2_ treatment in the MLN and HBO_2_ *per se* increased its level in spleen, impaired lymphocyte function was not observed.

Intensive research on the beneficial wound healing effects of HBO_2_ revealed its capacity to induce neovascularization, reduce oedema, decrease leukocyte adhesion, stimulate fibroblast expansion, and inhibit bacterial growth [14, 57]. Some of these processes are transcriptionally regulated by* HIF-1α*, namely, the vascular endothelial growth factor (VEGF) expression [58], regulatory T lymphocyte differentiation [59], and preservation of epithelial thigh junction integrity [60]. In addition, previous studies employing conditional deletion of epithelial* HIF-1α* or pharmacologic activation of* HIF-1α* in a murine model of colitis demonstrated a protective role for* HIF-1α* in colitis [61]. It has also been shown that* HIF-1* increases expression of barrier-protective genes (multidrug resistance gene-1, intestinal trefoil factor,* CD73*) [62], decreases* TNFα* mRNA expression [61], and enhances antimicrobial activity by transcribing beta-defensin 1 [63]. In the present study we found increased expression of* HIF-1α* gene in inflamed colonic tissue, and HBO_2_ further increased its level. In the peripheral lymphoid organs* HIF-1α* gene expression was changed (upregulated) by the inflammation while HBO_2_ treatment showed a tendency to reverse this increase. These data suggest involvement of different mechanisms controlling* HIF-1α* gene expression at the site of inflammation (colon) and the peripheral lymphoid organs (MLN and spleen), responsible for the initiation of the immune response and the T/B-cell expansion and differentiation, respectively. We also demonstrated a strong positive correlation between* HIF-1α* and* GPx1* mRNA levels in the colon (Figure 4(b)). This is in line with* in vitro* studies where overexpressed* HIF-1α* in colorectal cancer cells resulted in enhanced* GPx1* expression through TGF-*β*RI/Smad2/ERK1/2/*HIF-1α* signalling cascade, suggesting transcriptional regulation of* GPx1* by* HIF-1α* [64].

In the present study we found strong negative correlation between* HIF-1α* and* SOD1* mRNA expression. This is in accordance with a previous study showing that docosahexaenoic acid downregulates* SOD1* gene transcription through an HRE-mediated mechanism (HRE, hypoxia-response element), involving HIF signalling in human cancer cells [65]; thus our results indicate similar mechanism involved in SOD1 control in the murine colon mucosa* in vivo* during colitis and HBO_2_.

Previous studies revealed that* HIF-1α* mediated transcriptional regulation of different proinflammatory cytokines and growth factor genes are tissue and cell specific and include regulation trough alternative splicing, mRNA stability, and interactions with other transcription factors like NF-*κ*B [66–69]. In our study we found a strong correlation between* HIF-1α* and* IL-6* mRNA levels suggesting involvement of* HIF-1α* in transcriptional regulation of* IL-6* gene during colonic inflammation and HBO_2_.

**Figure 7:**
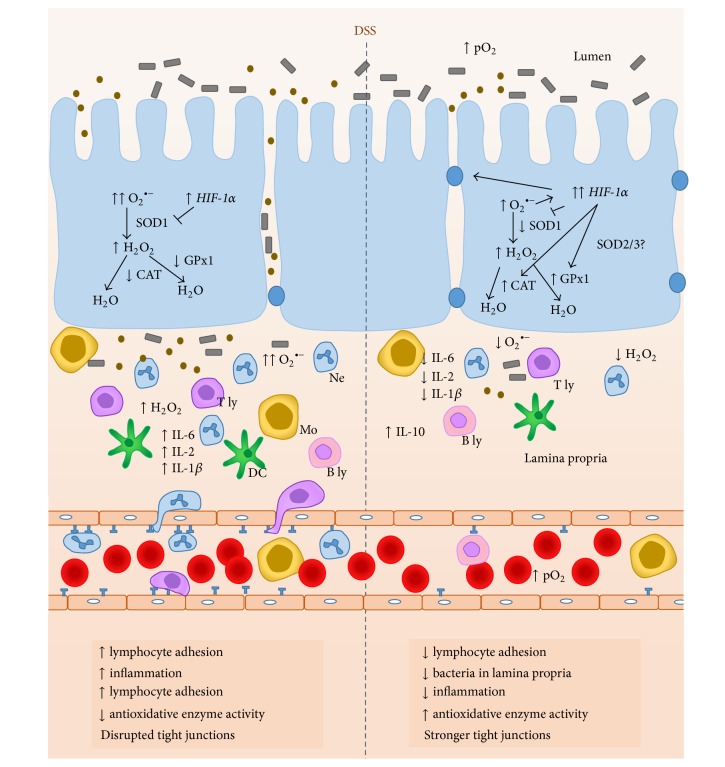
Proposed mechanisms mediating effects of HBO_2_ on the inflammation during DSS-induced colitis. Compared to untreated DSS-induced colitis (left of the vertical dotted line), high partial pressure of oxygen during HBO_2_ (right) activates* HIF-1α* signaling which leads to higher* CAT* and* GPx1* mRNA expression resulting in reduced oxidative stress in the inflamed colonic mucosa.* HIF-1α* mediated (negative) transcriptional regulation of proinflammatory cytokine IL-6 and reduced oxidative tissue injury leads to decreased neutrophil, monocyte, and lymphocyte adhesion and infiltration to the gut mucosa. In addition, HBO_2_ activates genes encoding for barrier-protection and genes responsible for improving tight junction integrity, all together resulting in reduced number of bacteria entering the lamina propria of gut mucosa. T ly, T lymphocyte; B ly, B lymphocyte; DC, dendritic cell; Ne, neutrophil; Mo, monocyte/macrophage;* HIF-1α*, hypoxia inducible factor 1 alpha; CAT, catalase; GPx1, glutathione peroxidase 1; SOD1, superoxide dismutase 1; pO_2_, partial O_2_ pressure; ↑, increased; ↓, decreased.

In conclusion, our results confirmed that HBO_2_ exerts an anti-inflammatory effect on DSS-induced colitis in mice, and this effect at least involves* HIF-1α* and antioxidative genes expression regulation (as outlined in Figure 7). However, further studies are necessary to identify the cells that may contribute to or are influenced by the effects upon HBO_2_ treatment.

## Supplementary Material

Supplementary Figure 1: Relative mRNA expression of VEGF (A), and PGK1 gene (B) in colon and their correlation to the HIF-1*α* gene expression. Relative mRNA expression was determined by real-time PCR, and the measured genes were normalized to the HPRT1 gene expression. BALB/c mice at the age of 10–12 weeks were randomly assigned into 4 groups (*n* = 5/group/experiments) CTRL—control mice, CTRL+HBO2—control mice undergoing HBO2 (60 min/2.4 ATM, 2x/day, days 1–8), DSS—mice receiving dextran sodium sulphate (DSS, 5% w/v, days 1–7), and DSS+HBO2—DSS treated mice undergoing HBO2. Data are presented as mean ± s.e.m. of two independent experiments, each with min. 5 mice/group. ∗statistically different from CTRL, *P* < 0.05; ^†^statistically different from CTRL+HBO2, *P* < 0.05.

## Figures and Tables

**Table 1:** Primer sequences, PCR product length, and primers annealing temperature used for qPCR analysis.

Gene		Sequence	PCR product length/bp	Annealing temperature/°C
*HIF-1α*	For	TGACGGCGACATGGTTTACA	280	63
	Rev	AATATGGCCCGTGCAGTGAA		

*SOD1*	For	GGAAGCATGGCGATGAAAGC	80	56
	Rev	GCCTTCTGCTCGAAGTGGAT		

*GPx1*	For	TCCAGTATGTGTGCTGCTCG	249	63
	Rev	GTGTCCGAACTGATTGCACG		

*CAT*	For	GGTGCCCCCAACTATTACCC	141	61
	Rev	GAATGTCCGCACCTGAGTGA		

*IL-1β*	For	GCCTTGGGCCTCAAAGGAAAGAATC	282	66
	Rev	GGAAGACACAGATTCCATGGTGAAG		

*IL-2*	For	CTCTGCGGCATGTTCTGGAT	163	65
	Rev	AGAAAGTCCACCACAGTTGCT		

*IL-6*	For	GCTGGAGTCACAGAAGGAGTGGC	117	63
	Rev	GGCATAACGCACTAGGTTTGCCG		

*HPRT1*	For	TCAGTCAACGGGGGACATAAA	142	59
	Rev	GGGGCTGTACTGCTTAACCAG		

**Table 2:** Catalase (CAT), glutathione peroxidase (GPx), and superoxide dismutase (SOD) activity in the colon, MLN, and spleen during colitis and HBO_2_ treatment.

Enzymatic activity (U mg^−1^ P)	Tissue	CTRL	CTRL + HBO_2_	DSS	DSS + HBO_2_
CAT	Colon	6.77 ± 3.04	5.39 ± 1.07	4.11 ± 0.33	4.23 ± 0.20
	MLN	13.90 ± 0.39	11.78 ± 1.71	9.06 ± 0.22^*∗*^	12.62 ± 0.42
	Spleen	10.44 ± 1.94	14.00 ± 0.97	6.75 ± 0.53^*∗*^	14.28 ± 1.11^#^

GPx	Colon	0.084 ± 0.028	0.097 ± 0.012	0.080 ± 0.008	0.072 ± 0.003
	MLN	0.164 ± 0.006	0.158 ± 0.021	0.175 ± 0.004	0.172 ± 0.007
	Spleen	0.098 ± 0.005	0.109 ± 0.003	0.114 ± 0.007	0.134 ± 0.004^*∗*†^

SOD	Colon	15.40 ± 3.29	27.86 ± 3.55^*∗*^	30.56 ± 2.70^*∗*^	30.97 ± 1.58^*∗*^
	MLN	36.53 ± 2.80	33.96 ± 3.33	34.48 ± 1.62	35.30 ± 1.75
	Spleen	19.87 ± 1.15	17.91 ± 0.72	20.93 ± 1.03	25.34 ± 1.61^*∗*†^

BALB/c mice at the age of 10–12 weeks were randomly assigned into 4 groups (*n* = 5/group): CTRL: control mice, CTRL + HBO_2_: control mice undergoing HBO_2_ (60 min/2.4 ATM, 2x/day, days 1–8), DSS: mice receiving DSS (5% w/v, days 1–7), and DSS + HBO_2_: DSS treated mice undergoing HBO_2_. Presented data (mean ± s.e.m.) are representative results from one experiment with min. five mice/group; *P* < 0.05 was considered significant; ^*∗*^statistically different from CTRL, *P* < 0.05; ^#^statistically different from DSS, *P* < 0.05; ^†^statistically different from CTRL + HBO_2_, *P* < 0.05.

## Abbreviations

CAT: Catalase
CTRL: Control
DAI: Disease activity index
DCF DA: Dichlorofluorescein acetate
DSS: Dextran sodium sulfate
GPx1: Glutathione peroxidase 1
HBO_2_: Hyperbaric oxygen
HRE: Hypoxia-response element
*HIF-1α*: Hypoxia inducible factor-1 alpha
IBD: Inflammatory bowel disease
MFI: Median fluorescence intensity
MLN: Mesenteric lymph node
O_2_^∙−^: Superoxide radical
ONOO^−^: Peroxynitrite
PMA: Phorbol 12-myristate 13-acetate
RNS: Reactive nitrogen species
ROS: Reactive oxygen species
s.e.m.: Standard error mean
SOD1: Superoxide dismutase 1
TCR: T cell receptor
Treg: T regulatory lymphocyte
VEGF: Vascular endothelial growth factor.
