# Supplementary figures and images for: Anti-Inflammatory Effects of Hyperbaric Oxygenation during DSS-Induced Colitis in BALB/c Mice Include Changes in Gene Expression of HIF-1α, Proinflammatory Cytokines, and Antioxidative Enzymes
Source: Mediators Inflamm. 2016 Aug 30;2016:7141430. doi: 10.1155/2016/7141430 (PMC5021505; doi:10.1155/2016/7141430)

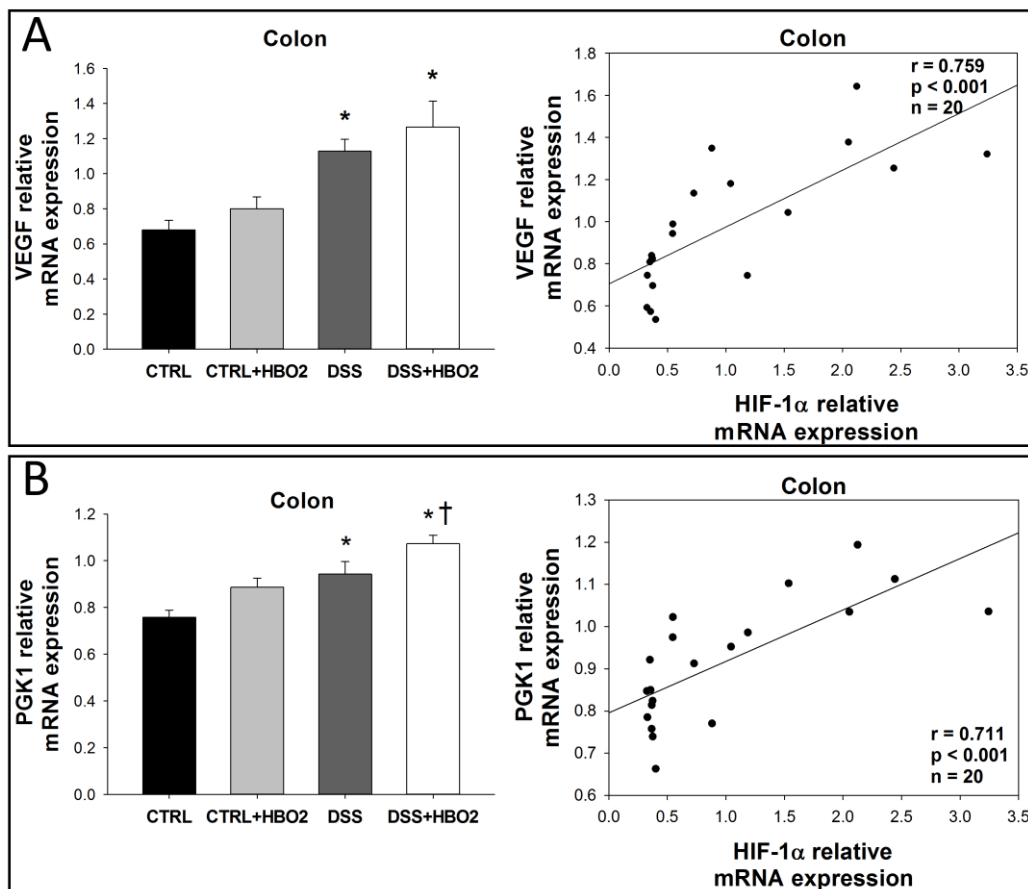

**Supplementary figure 1.**

Supplement: Supplementary file 1 — Supplementary Figure 1: Relative mRNA expression of VEGF (A), and PGK1 gene (B) in colon and their correlation to the HIF-1α gene expression. Relative mRNA expression was determined by real-time PCR, and the measured genes were normalized to the HPRT1 gene expression. BALB/c mice at the age of 10–12 weeks were randomly assigned into 4 groups (n = 5/group/experiments) CTRL—control mice, CTRL+HBO2—control mice undergoing HBO2 (60 min/2.4 ATM, 2x/day, days 1–8), DSS—mice receiving dextran sodium sulphate (DSS, 5% w/v, days 1–7), and DSS+HBO2—DSS treated mice undergoing HBO2. Data are presented as mean ± s.e.m. of two independent experiments, each with min. 5 mice/group. ∗statistically different from CTRL, P < 0.05; †statistically different from CTRL+HBO2, P < 0.05. [file 7141430.f1.pdf]
